# Enhanced ubiquitin-dependent degradation by Nedd4 protects against α-synuclein accumulation and toxicity in animal models of Parkinson's disease

**DOI:** 10.1016/j.nbd.2013.12.011

**Published:** 2014-04

**Authors:** Sian E. Davies, Penelope J. Hallett, Thomas Moens, Gaynor Smith, Emily Mangano, Hyoung Tae Kim, Alfred L. Goldberg, Ji-Long Liu, Ole Isacson, George K. Tofaris

**Affiliations:** a Nuffield Department of Clinical Neurosciences, University of Oxford, UK; b Oxford Parkinson's Disease Centre, University of Oxford, UK; c MRC Functional Genomics Unit, Department of Physiology, Anatomy and Genetics, University of Oxford, UK; d Neuroregeneration Research Institute, McLean Hospital, Harvard Medical School, USA; e Department of Cell Biology, Harvard Medical School, USA

**Keywords:** Lysosome, Ubiquitination, Protein-degradation, Endosomal-trafficking

## Abstract

Parkinson's disease is a neurodegenerative disorder, characterized by accumulation and misfolding of α-synuclein. Although the level of α-synuclein in neurons is fundamentally linked to the onset of neurodegeneration, multiple pathways have been implicated in its degradation, and it remains unclear which are the critical ubiquitination enzymes that protect against α-synuclein accumulation in vivo. The ubiquitin ligase Nedd4 targets α-synuclein to the endosomal–lysosomal pathway in cultured cells. Here we asked whether Nedd4-mediated degradation protects against α-synuclein-induced toxicity in the *Drosophila* and rodent models of Parkinson's disease. We show that overexpression of Nedd4 can rescue the degenerative phenotype from ectopic expression of α-synuclein in the *Drosophila* eye. Overexpressed Nedd4 in the *Drosophila* brain prevented the α-synuclein-induced locomotor defect whereas reduction in endogenous Nedd4 by RNAi led to worsening motor function and increased loss of dopaminergic neurons. Accordingly, AAV-mediated expression of wild-type but not the catalytically inactive Nedd4 decreased the α-synuclein-induced dopaminergic cell loss in the rat substantia nigra and reduced α-synuclein accumulation. Collectively, our data in two evolutionarily distant model organisms strongly suggest that Nedd4 is a modifier of α-synuclein pathobiology and thus a potential target for neuroprotective therapies.

## Highlights

• Nedd4 rescues the α-synuclein-induced rough-eye phenotype.
• Nedd4 prevents the α-synuclein-induced locomotor defect in the *Drosophila* brain.
• Overexpressed Nedd4 promotes α-synuclein degradation in vivo.
• Nedd4 knockdown increases the α-synuclein-induced loss of dopaminergic neurons.
• Nedd4 decreases dopaminergic cell loss in the rat substantia nigra.

## Introduction

Parkinson's disease (PD) is a common, age-related neurodegenerative disorder without cure. Although primarily a movement disorder, the disease process is eventually widespread causing dementia and other non-motor symptoms. This relentless clinical course is accompanied by neuronal loss and intracellular accumulation of misfolded α-synuclein in the dopaminergic neurons of the substantia nigra (SN) and other brain regions (Tofaris and Spillantini, 2007). Treatments that aim to slow down or stop neurodegeneration in PD are lacking. There is now extensive evidence indicating that α-synuclein content in neurons is critical for the onset of neurodegeneration; familial cases with *α-synuclein* gene multiplications show a strong gene-dosage effect (Ross et al., 2008) and *α-synuclein* promoter polymorphisms that increase protein expression are a risk factor for PD (Maraganore et al., 2006). In animal studies, overexpression of human α-synuclein leads to degeneration of nerve terminals and locomotor defects (Tofaris et al., 2006; Chung et al., 2009a, 2009b) and such changes can be reversed when the transgene is switched off (Lim et al., 2011). Conversely, knockout of α-synuclein in mice confers protection against certain toxins (Dauer et al., 2002). Therefore, promoting the degradation of α-synuclein and thus reducing its basal level inside neurons could represent a novel neuroprotective strategy for PD. Such strategy necessitates the identification of enzymes that directly catalyze the clearance of α-synuclein and demonstration that boosting their activity is neuroprotective in animal models. Proteins destined for selective degradation are conjugated to a ubiquitin chain in a three-step catalytic process which involves a ubiquitin-activating enzyme E1, a ubiquitin conjugating enzyme E2 and a ubiquitin ligase (E3). There are more than 650 E3s, which regulate substrate specificity and different types of ubiquitin linkages, which determine whether the protein is degraded by the proteasome or the lysosome. Although both proteasomes and lysosomes can degrade α-synuclein, the latter have recently emerged as especially important in PD pathogenesis (Tofaris, 2012). We have previously shown that Nedd4 (neuronally-expressed developmentally down-regulated gene 4) is a ubiquitin ligase for α-synuclein (Tofaris et al., 2011). Since Nedd4 targets substrates to lysosomal degradation, we hypothesized that it is protective against α-synuclein toxicity in relevant animal models of PD.

## Materials and methods

### Drosophila genotypes

The *UAS-α-synuclein* was obtained from M. Feany (Harvard Medical School) and the *UAS-dNedd4* line was obtained from M. Baron (Manchester University). *UAS-dNedd4-RNAi 13121* was obtained from the Vienna Drosophila RNAi Center. The GAL4 UAS expression system was used to overexpress these transgenes either pan-neuronally at 25 °C using an *Elav*-GAL4 driver, or specifically in the eye using *GMR*-GAL4 at 25 and 28 °C. Driver lines were crossed to *w^1118^* to generate heterozygous controls. The following genotypes were used: (1) *Elav*-GAL4/+; (2) *Elav*-GAL4/+; *UAS-α-synuclein*/+; (3) *Elav*-GAL4/+; *UAS-Nedd4 RNAi 13121*/+; (4) *Elav*-GAL4/+; *UAS-dNedd4*/+; (5) *Elav*-GAL4/+; *UAS-α-synuclein*/+; *UAS-Nedd4 RNAi 13121*/+; (6) *Elav*-GAL4/+; *UAS-α-synuclein*/+; *UAS-dNedd4*/+; (7) *GMR*-GAL4/+; (8) *UAS-α-synuclein*/+; *GMR*-GAL4/+; (9) *UAS-Nedd4 RNAi 13121*/*GMR*-GAL4; (10) *UAS-dNedd4*/*GMR*-GAL4; (11) *UAS-α-synuclein*/+; *UAS-Nedd4 RNAi 13121*/*GMR*-GAL4; (12) *UAS-α-synuclein*/+; *UAS-dNedd4*/*GMR*-GAL4. Flip-out mitotic clones were generated using the inducible GAL4 driver, HsFlp, UAS-GFPnls; UAS-Dcr2; tub>GAL80>GAL4/SM5, Cyo-TM6, Tb. The >GAL80> cassette is recombined out following heat-shock, thereby inducing tub-GAL4 driven expression of genes under the control of the UAS promoter. The lines generated were: (1) HsFlp, UAS-GFP_nls_/+; UAS-α-synuclein/UAS-Dcr2; tub>GAL80>GAL4/+; (2) HsFlp, UAS-GFP_nls_/+; UAS-Dcr2/+; tub>GAL80>GAL4/UAS-Nedd4-RNAi; and (3) HsFlp, UAS-GFP_nls_/+; UAS-Dcr2/+; tub>GAL80>GAL4/UAS-dNedd4. These progeny were heat-shocked for 1 h at 37 °C at 24 h and 48 h after egg deposition.

### Scanning electron microscopy of the *Drosophila* eye

Age-matched, male flies were fixed in 70% ethanol. The flies were then dehydrated in 100% ethanol, dried and mounted on SEM stubs. The samples were then sputter coated with gold and imaged using a JEOL JSM 6390 scanning electron microscope. To quantitate the eye phenotype, we randomly selected 20 flies per genotype, which were scored by two blinded assessors independently using objective features of severity that were adapted from Pandey et al. (2007). Eyes were examined for abnormal bristle orientation, ommatidial fusion or pitting and disorganization of the ommatidial array, and given one point if this feature was present, 2 points if the affected area involved more than 5% of the eye and 4 points if the affected area involved more than 25% of the eye.

### Immunofluorescence and confocal microscopy for *Drosophila* eyes

Larval eye discs were fixed for 10 min in 4% paraformaldehyde. Primary antibodies used were: mouse anti-α-synuclein (BD Transduction, 1:500) and rabbit anti-Nedd4 (Abcam, 1:500). The Cy5 conjugated (Jackson ImmunoResearch, 1:1000) secondary antibody was used. Samples were imaged using a Zeiss LSM 510 META confocal microscope.

### Immunoblotting of *Drosophila* brain lysates

For each genotype 5 male adult heads were homogenized in lysis buffer (150 mM NaCl, 10 mM Tris pH7.4, 1% Triton-X, 0.1% SDS and complete protease inhibitor cocktail, SIGMA). Samples were quantified and loaded on a NuPAGE 10–12% Bis–Tris gel (Invitrogen). The following primary antibodies were: mouse anti-tubulin (Developmental Studies Hybridoma Bank, 1:1000), mouse anti-α-synuclein, Syn1 (BD Transduction, 1:400), rabbit anti-Nedd4 (Abcam, 1:5000), and rabbit anti-actin (Sigma, 1:1000). Blots were visualized using HRP-conjugated secondary antibodies and the ECL Detection Reagent (Amersham).

### Climbing assays

Locomotor function was assayed using a startle-induced negative geotaxis assay and the climbing ability of the flies measured under red light (adapted from Feany and Bender, 2000). Up to 10 flies were placed per vial and tapped 3 times. For each line between 3 and 5 vials were included in each experiment. The number of flies above a predetermined line was recorded after 4 s of climbing. All lines were crossed and maintained at 25 °C with a 12 h light/dark cycle. Similar results were obtained from two independently derived transgenic lines.

### Viability assays

40 male flies of each genotype were incubated at 25 °C in vials containing standard media. Each day *Drosophila* were transferred to fresh vials, and the number of flies which had died was recorded. At the end of the experiment, the remaining flies were counted.

### AAV2 nigral injections in rats

The AAV2/2-A53T α-synuclein and green fluorescent protein (GFP) vectors were prepared as previously described (Chung et al., 2009a, 2009b). The AAV2/2-Nedd4 vector consists of full-length rat wild-type, or a Cys → Ser point mutation in the HECT domain catalytic site. Both Nedd4^WT^ and Nedd4^C-S^ constructs were epitope-tagged with T7 at the N-terminus and under the control of the human synapsin promoter. The final titer used for injection of the vectors was 2 × 10^12^ genome copies/ml. Animals were housed according to standard conditions, in a dark/light cycle of 12 h, with ad libitum access to food and water. Female Sprague–Dawley rats weighing ~ 250 g (Charles River Laboratories) were used in the animal experiments and injected as previously described (Chung et al., 2009a, 2009b). These experiments had previously been approved by the McLean Hospital Institutional Animal Care and Use Committee. Stereotaxic coordinates for the surgeries were taken from the “Rat Atlas” by Paxinos and Watson (1986). Before surgery, the animals were anesthetized with xylazine and ketamine (3 mg/kg and 60 mg/kg, respectively). The animals were placed in a stereotaxic frame (Stoelting), where a 10 μl Hamilton syringe and 31 gauge needles were used as a delivery system. All injections were made into the substantia nigra using the following anteroposterior (AP), mediolateral (ML), and dorsoventral (DV) coordinates: first site, AP = − 4.8 mm; ML = − 2.0 mm; DV = − 7.2 mm relative to dura, second site, AP = − 5.5 mm; ML = − 1.9 mm, DV = 7.0 mm relative to dura; toothbar (TB) set at − 3.3 mm. For coinjection of AAV-A53T α-synuclein with either AAV Nedd4^WT^ or AAV Nedd4^C-S^, viruses were mixed in the same infusate at a final titer of 2 × 10^12^ genome copies/ml. Two microliters of either A53T α-synuclein + AAV Nedd4^WT^, A53T α-synuclein + AAV Nedd4^C-S^, or AAV GFP control transgene was injected per site at a rate of 0.5 μl/min using microinfusion pumps (Stoelting), with a wait time of 10 min between injection. At 19 weeks post-AAV injection, animals were terminally anesthetized and perfused as detailed in Chung et al. (2009a, 2009b).

### Immunohistochemistry on rat brain sections

Immunohistochemistry was performed on serial free-floating sections using the following primary antibodies: rabbit anti-tyrosine hydroxylase (TH) (Pel Freez; 1:300), mouse anti-human α-synuclein (LB509, Zymed, 1:500) and anti-goat T7 (AbCam, 1:200). Confocal analysis was performed using a Zeiss LSK510/Meta station.

### Stereology on rat brain sections

Unbiased quantification of TH/NeuN-positive neuronal numbers within the SNc and ventral tegmental area (VTA) was performed by investigators blind to group treatments using a Zeiss Axiovert microscope coupled to an Optronics Microfire digital camera for visualization of tissue sections, and Stereo Investigator software, and using stereologic principles as previously described (Chung et al., 2009a, 2009b). Briefly, TH/NeuN-positive neurons of the injected SN and VTA were counted under brightfield illumination at 40 × magnification and the total number of TH/NeuN-positive neurons was estimated from coded slides using the optical fractionator method. The anterior and posterior boundaries of the SN included in the analysis were defined according to the area transduced by AAV2-GFP in preliminary experiments [approximately − 4.80 mm through − 6.00 mm from bregma (according to the rat brain atlas of Paxinos and Watson, 1986)]. The coefficients of error were calculated according to the procedure of West (1993) and values < 0.10 were accepted.

### Biochemical fractionation of the rat brain tissue

Tissue was weighed and homogenized in 30 volumes of lysis buffer (150 mM NaCl, 10 mM Tris pH7.4 and complete protease inhibitor cocktail from SIGMA). All centrifugation steps were performed at 4 °C. Homogenates were initially centrifuged at 1000 *g* for 5 min to remove debris and nuclei and the resultant supernatants from this step were centrifuged at 120,000 *g* for 30 min. The supernatant was designated as the cytosolic fraction and the pellet was extracted in RIPA buffer (150 mM NaCl, 10 mM Tris pH7.4, 1% Triton-X, 0.1% SDS and complete protease inhibitor cocktail) and centrifuged at 120,000 *g* for 30 min. The resulting pellet was solubilized in SDS and designated as RIPA-insoluble/SDS soluble fraction.

### Ubiquitination assay

Wild-type and A53T mutant human α-synuclein protein (untagged full-length) was added in excess to the ubiquitination mixture containing 6His-E1 (50 nM), UbcH5 (750 nM), Nedd4 (500 nM) and ubiquitin (59 mM) in a buffer composed of 20 mM Tris–HCl (pH7.6), 20 mM KCl, 5 mM MgCl2, 2 mM ATP, and 1 mM DTT.

### Statistics

For the climbing assay, the proportion of flies which climbed above a predetermined line were compared in order to account for different numbers of flies in some tubes caused by death or escape. Proportions were ArcSin root transformed and a one-way ANOVA was performed for each time point; when P < 0.05 a subsequent unplanned comparison using a Tukey–Kramer test was performed to determine which genotypes significantly differed from one another. For viability assays, log rank tests were performed to individually compare each genotype to elav-GAL4 controls, using a Bonferroni adjusted P-value (P = 0.0083) to adjust for multiple comparisons.

## Results

### Nedd4 rescues the α-synuclein-induced rough eye phenotype

**Fig. 1:**
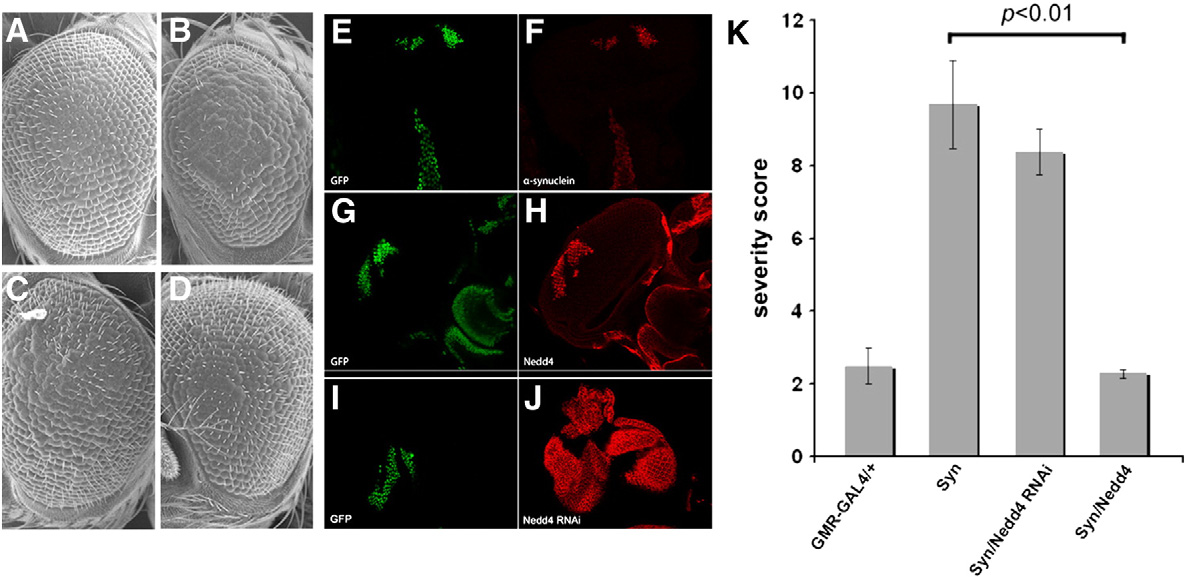
Eye-specific expression of α-synuclein by *GMR-GAL4* causes a rough eye phenotype that is prevented by Nedd4 co-expression. Unlike control flies expressing *GMR-GAL4/+* (A), overexpression of α-synuclein caused a rough eye phenotype (B) that is detectable only by SEM. (C) The phenotype was not worsened in doubly transgenic flies co-expressing Nedd4 RNAi and α-synuclein (labeled Syn/Nedd4 RNAi). (D) Overproduction of Nedd4 prevented the α-synuclein-induced degenerative eye phenotype (labeled Syn/Nedd4). Scale bar, 100 μm. ‘Flip-out’ clones confirmed that α-synuclein (E, F) Nedd4 (G, H) and Nedd4 RNAi (I, J) were readily expressed in GFP positive clones in larval eye discs upon heat shock. (K) Quantitative analysis of the severity of the eye phenotypes; *n* = 20 SEM images per group, p < 0.01 using ANOVA.

Nedd4 is a highly conserved E3 and essential for viability (Rotin and Kumar, 2009). We therefore first asked whether expression of wild-type or catalytically inactive Nedd4 or knockdown of the endogenous protein per se specifically in the *Drosophila* eye causes any phenotype. We found that Nedd4 overexpression or knockdown did not cause a phenotype whereas expression of catalytically inactive nedd4 was associated with toxicity (Suppl. Fig. 1). Because of this finding, we asked whether small changes in wild-type Nedd4 by overexpression or knockdown can modify the toxicity from ectopic expression of wild-type human α-synuclein. In this model, transgenic expression of toxic proteins causes cell degeneration, leading to a ‘rough eye’ phenotype (Pandey et al., 2007) that depending on severity is detectable by light or scanning electron microscopy (SEM). We used the *GMR-*GAL4 driver and incubated the transgenic flies at 25 °C and 28 °C to determine whether slight variations in protein expression levels can induce a phenotype. Using SEM, we found that at 28 °C, overexpression of α-synuclein induced a rough eye phenotype (Fig. 1 panels A, B), whereas concurrent over-production of Nedd4 (at 28 °C) rescued this defect (Fig. 1D). Knockdown of endogenous Nedd4 using transgenic expression of RNAi did not cause an obvious further worsening of the α-synuclein-induced phenotype (Fig. 1C). Quantification of the phenotype severity is shown in Fig. 1K and detailed in the methods. Nedd4 knockdown or overexpression by *GMR*-GAL4 per se did not cause an eye phenotype at 28 °C as detailed above. We confirmed the presence of α-synuclein, Nedd4 or Nedd4 RNAi transgenes in the eye, by generating flip-out clones in combination with immunofluorescence for α-synuclein or Nedd4 (Fig. 1 panels E–J).

### Neuronal Nedd4 levels modify the motor phenotype in *Drosophila* expressing α-synuclein

**Fig. 2:**
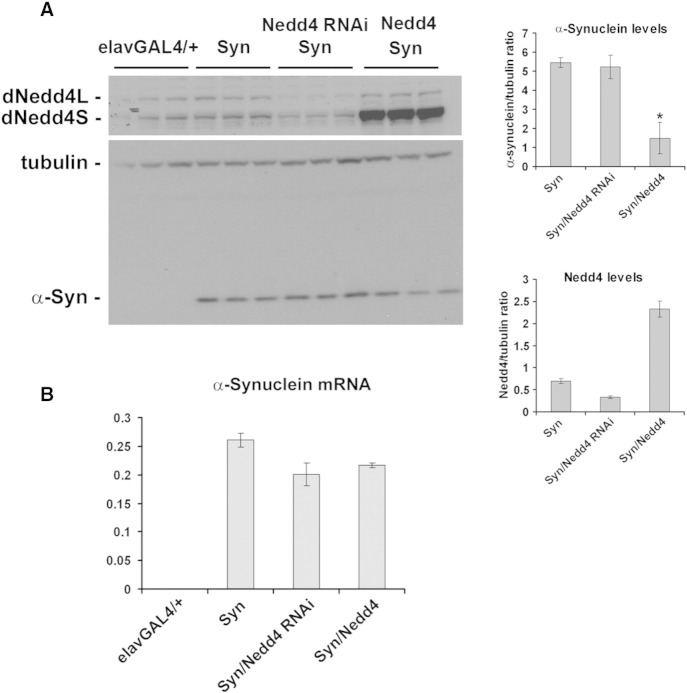
Increased neuronal Nedd4 reduces α-synuclein levels in the *Drosophila* brain. (A) Representative immunoblot with three independent samples from each transgenic line: *elav-*GAL4/+, α-synuclein flies (Syn) and doubly transgenic α-synuclein flies with either Nedd4 RNAi (labeled Syn/Nedd4 RNAi) or Nedd4 overexpression (labeled Syn/Nedd4). Quantitative band densitometry showed that, relative to tubulin loading control, neuronal α-synuclein was significantly reduced by 2-fold in flies co-expressing Nedd4 (upper histogram). Neuronal expression of Nedd4 was reduced by 2-fold in RNAi expressing flies and increased by 5–6 fold in flies expressing the short isoform of Nedd4 (lower histogram). Values represent mean ± SEM from three independent experiments. (B) Quantitative PCR did not show a significant difference in α-synuclein mRNA levels between the three transgenic lines and no signal was detected in the control genotype as expected.

**Fig. 3:**
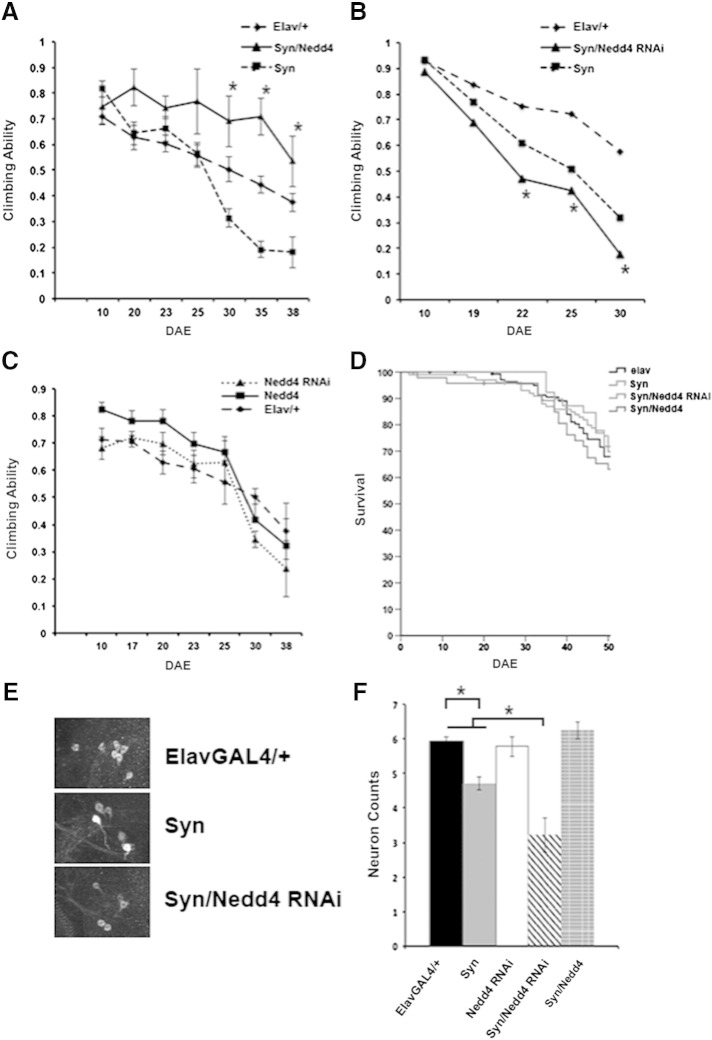
Nedd4 levels modify the α-synuclein-induced motor phenotype and dopaminergic cell number in *Drosophila*. (A) Accelerated loss of climbing ability was seen in transgenic flies expressing α-synuclein throughout the nervous system (*elav-*GAL4 driver). The climbing ability of doubly transgenic lines expressing Nedd4 and α-synuclein was significantly improved (shown by asterisks) when compared to α-synuclein expressing flies and was similar to the control genotype, *elav-*GAL4/+. (B) Unlike the result in the eye, reduction of endogenous Nedd4 specifically in the nervous system caused a worsening in the climbing scores, which were significantly different between doubly transgenic Nedd4 RNAi/α-synuclein compared to either α-synuclein or the control genotype, *elav-GAL4/+* (error bars are hidden within the symbols). (C) No significant difference was detected when Nedd4 per se was overexpressed or knocked-down. *p < 0.05 using ANOVA with subsequent Tukey–Kramer test. Values represent mean ± SEM. (D) Kaplan–Meier survival curves were similar at different days after eclosion (DAE). (E) Dopaminergic neurons in the dorsomedial cluster were visualized with anti-TH antibodies and counted. (F) A significant loss of dopaminergic neurons was seen in Nedd4 RNAi/α-synuclein doubly transgenic flies when compared to α-synuclein or *elav-GAL4/+* phenotypes. Co-expression of Nedd4 was protective against α-synuclein-induced cell loss (n = 10 brains/group). *p < 0.05 using ANOVA with subsequent Tukey–Kramer test. Values represent mean ± SEM.

Our findings in the *Drosophila* eye strongly argue that boosting Nedd4 activity is required to mitigate the toxic phenotype from increased ectopic expression of α-synuclein. Although an indirect mechanism of protection cannot be excluded, our previous in vitro findings (Tofaris et al., 2011), suggest that at least in part, Nedd4-mediated protection is due to enhanced degradation of α-synuclein in vivo. To test this prediction, we expressed wild-type Nedd4 or its RNAi pan-neuronally using the *elav-GAL4* driver. *Drosophila* Nedd4 has two spliced variants, a short isoform Nedd4S (~ 92 kDa), and a longer isoform Nedd4L (~ 112 kDa) (Zhong et al., 2011). Both contain the C2 domain, three WW domains which recognize proline-rich sequences on protein-substrates and the catalytically active HECT domain. Previous studies showed that ubiquitous expression of the short isoform does not affect larval development and rescues the phenotype of Nedd4 mutants in *Drosophila*, whereas overexpression of the long isoform causes lethality (Zhong et al., 2011). We thus generated double transgenic flies co-expressing Nedd4 RNAi or the short isoform of *Drosophila* Nedd4 with α-synuclein and measured the level of ectopically expressed α-synuclein in total brain lysates in single and doubly transgenic flies. These experiments showed that when Nedd4 levels were increased by 5–6 fold, a significant 2-fold reduction in neuronal α-synuclein was detected (Fig. 2A and Suppl. Fig. 2). To confirm that changes in α-synuclein protein levels were not due to promoter competition or variable mRNA levels, we performed qPCR in these lines. These data showed that α-synuclein mRNA is not significantly different between transgenic lines and were undetected in the *elav-GAL4/+* control flies as expected (Fig. 2B). To test whether enhanced Nedd4-mediated degradation of α-synuclein specifically in brain affords protection and validate our earlier observations in the eye using an alternative read-out of toxicity, we then measured the climbing ability of transgenic flies at different time-points after eclosion. This climbing response was previously shown to reflect an age-related motor dysfunction due to α-synuclein expression and thus a measure of α-synuclein-induced toxicity (Chen et al., 2009; Feany and Bender, 2000). Strikingly, we found that Nedd4 overexpression prevented the climbing deficit that is associated with α-synuclein expression (Fig. 3A) whereas Nedd4 knockdown significantly worsened this climbing response in contrast to the knockdown effect on the eye phenotype (Fig. 3B). Overexpression or knockdown of Nedd4 per se did not alter the climbing ability when compared to *elav-GAL4* controls at the same (late) time points after eclosion (Fig. 3C). In addition, we assessed lifespan and did not find any significant difference in the transgenic lines that could account for the difference in the climbing performance (Fig. 3D).

Although the total level of α-synuclein as assessed by immunoblotting, was not increased in brain lysates when Nedd4 was knocked down by 2-fold in neurons, the differential effect of Nedd4 RNAi in the motor but not the eye phenotype, suggests that endogenous as opposed to overexpressed Nedd4 may be critical for a subpopulation of neurons that are especially vulnerable to α-synuclein toxicity. To address this issue, we identified and counted the number of dopaminergic neurons in the dorsomedial cluster, which were previously shown to be susceptible to α-synuclein toxicity (Auluck et al., 2002; Feany and Bender, 2000). Our data showed that the combination of α-synuclein overexpression and Nedd4 knocked-down, led to a significant reduction in the number of TH-positive neurons in the dorsomedial cluster when compared to controls, α-synuclein or Nedd4 RNAi expression alone (Figs. 3E, F). In contrast, co-expression of Nedd4 prevented the α-synuclein-induced dopaminergic cell loss (Figs. 3E, F). Thus, although Nedd4 overexpression is generally protective against α-synuclein toxicity, the endogenous protein is critical in vulnerable neuronal subpopulations.

### Overexpression of wild-type but not the catalytically inactive Nedd4 decreased dopaminergic cell loss in the rat model of α-synucleinopathy

**Fig. 4:**
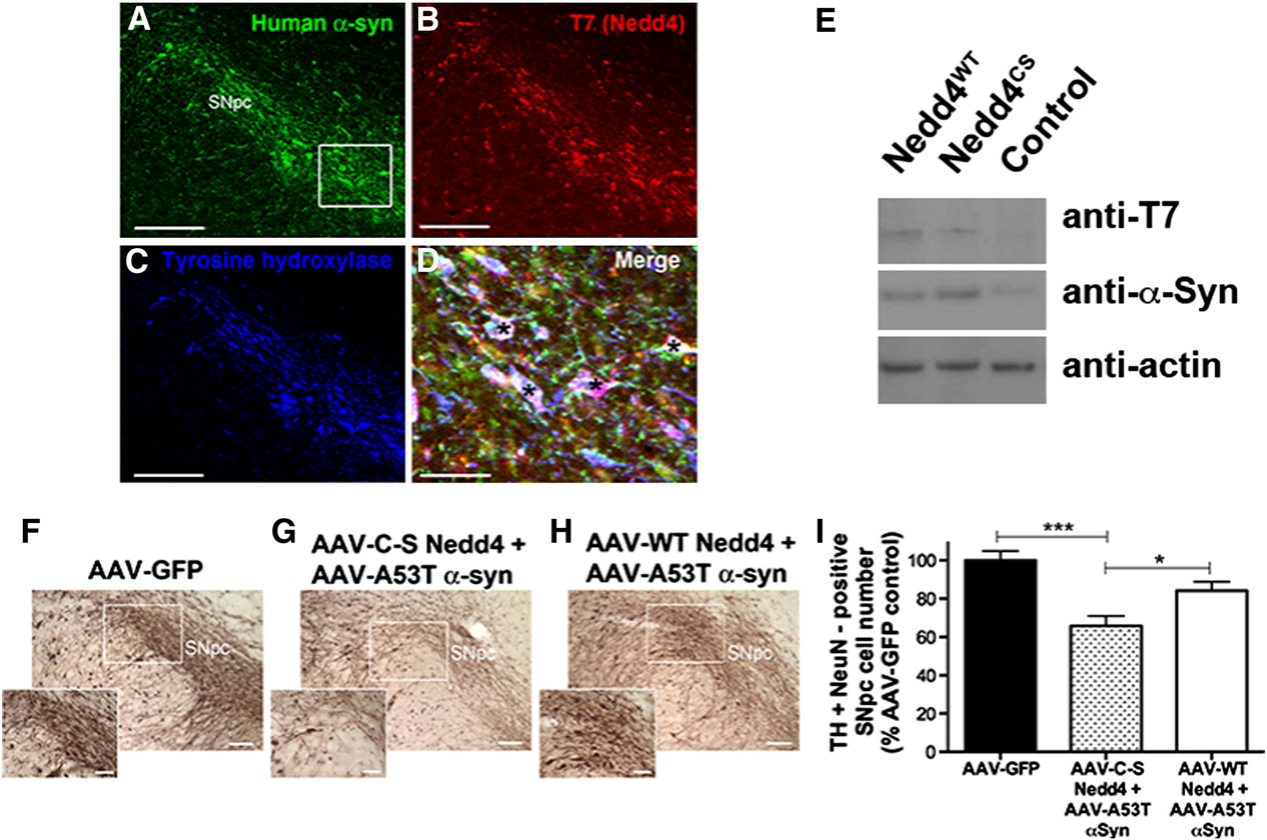
Overexpression of wild-type but not catalytically inactive Nedd4 prevents α-synuclein-induced loss of dopaminergic neurons. Virally-mediated overexpression of human A53T α-synuclein at 19 weeks post-AAV injection was confirmed using the LB509 antibody (panel A), and Nedd4 overexpression was verified using anti-T7 (panel B). TH was used to label dopaminergic neurons (panel C). Co-expression of both Nedd4 and human α-synuclein within TH-immunoreactive SNc neurons is shown in panel D, and represents the boxed area illustrated in panel A. Examples of TH-immunoreactive neurons expressing both transgenes are marked with asterisks. Expression was also confirmed by immunoblotting of SN lysates at 19 weeks with antibodies against α-synuclein (Syn1) and T7 for Nedd4 (panel E). Representative images and high magnification insets are shown in panels F–H for TH/NeuN doubly labeled neurons in the SNc for each group. (I) When compared to AAV-GFP controls (n = 5), there was a significant reduction in the number of dopaminergic neurons in rats expressing Nedd4^C-S^ and A53T α-synuclein (n = 8) whereas co-expression of Nedd4^WT^ (n = 7) afforded protection. *p < 0.05, p < 0.001 (one-way ANOVA, Tukey's multiple comparison post-hoc test). Scale bar, panels A, B = 200 μm, panel D = 50 μm, panels F–G = 100 μm, insets = 50 μm.

**Fig. 5:**
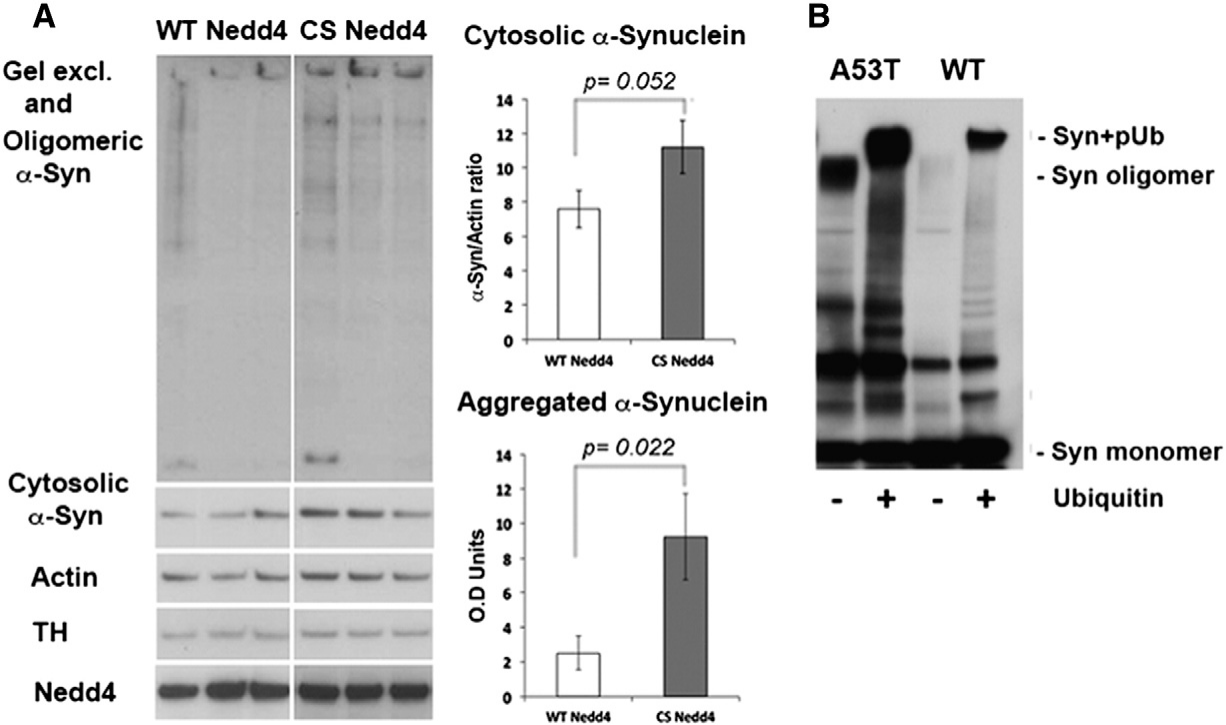
Wild-type but not catalytically inactive Nedd4 prevents α-synuclein accumulation in the rat SN. The level of cytosolic and aggregated α-synuclein was quantified following serial fractionation and immunoblotting of SN lysates at 19 weeks post-AAV injection of either A53T α-synuclein/Nedd4^WT^ or A53T α-synuclein/Nedd4^C-S^. (A) Representative immunoblot showing cytosolic and aggregated α-synuclein at 19 weeks post-AAV injection for three animals per group. Actin is shown as loading control. Quantitative band densitometry (*n* = 5–6 animals per group) showed that the cytosolic α-synuclein content was reduced by 33% in rats co-expressing Nedd4^WT^ (panel A, upper histogram) whereas the RIPA-insoluble/SDS soluble (gel excluded and oligomeric) α-synuclein, was significantly increased by 3.6-fold in rats co-expressing Nedd4^C-S^ (panel A, lower histogram). B. Recombinant wild-type and A53T mutant α-synuclein was incubated with purified Nedd4, UbcH5 as the E2 with or without ubiquitin, and ubiquitination was assayed using anti-α-synuclein antibodies. Polyubiquitination (syn + pUb) of both recombinant WT and A53T mutant α-synuclein by Nedd4 was detected. High molecular weight species of aggregated A53T α-synuclein, present in the reaction before ubiquitin was added, were also ubiquitinated by Nedd4 as evidenced by the increase in their molecular weight upon addition of recombinant ubiquitin. Monomeric α-synuclein was added in excess and thus not clearly depleted in this reaction.

To test the role of Nedd4 activity in dopaminergic neurons further and assess the neuroprotective function of Nedd4 in a mammalian animal model, we asked whether wild-type (Nedd4^WT^) or catalytically inactive (active site cysteine mutated to serine, Nedd4^C-S^) Nedd4 expression prevents cell death from virally-mediated expression of the human A53T mutant α-synuclein in dopaminergic neurons (Chung et al., 2009a, 2009b). Co-expression of both Nedd4 and α-synuclein was detected in the dopaminergic neurons (Fig. 4, panels A–E) and levels of Nedd4 overexpression, as detected by immunoblotting, were equal in the two lines (Figs. 4E and 5A). We have previously shown that AAV-mediated overexpression of human A53T α-synuclein on the synapsin promoter in rats produces a progressive degeneration of SN pars compacta (SNc) dopaminergic neurons (Chung et al., 2009a, 2009b). Although the extent of cell loss in this model (32% loss compared to GFP expressing values, Chung et al., 2009a, 2009b) is not sufficient to produce a clear behavioral phenotype or changes in striatal nerve terminals, we used this system to specifically examine the number of SNc dopaminergic neurons at 19 weeks post-AAV co-injection, a time-point in which there is significant neurodegeneration. Unbiased stereological quantification of neurons co-labeled for NeuN and TH showed that the number of nigral dopaminergic neurons in rats injected with α-synuclein and Nedd4^C-S^ was significantly reduced to 65.8% (± 5.2) of GFP control values (p < 0.001). Importantly, the number of NeuN and TH doubly labeled neurons co-injected with α-synuclein/Nedd4^WT^ (84.2 ± 4.6% of GFP control) was significantly increased compared to the α-synuclein/Nedd4^C-S^ group (p < 0.05) and not significantly different compared to GFP controls (p > 0.05) (Fig. 4, panels F–I). These data suggest that the ubiquitin ligase activity of Nedd4 is necessary to prevent α-synuclein-induced neurodegeneration in vivo. In control experiments we did not find significant differences in the extent of co-localization of either A53T α-synuclein and Nedd4^WT^, or A53T α-synuclein and Nedd4^C-S^ (90.3 ± 3.2% and 94.8 ± 2.2% of neurons respectively, p > 0.05). Triple co-localization of human α-synuclein, T7 for tagged Nedd4 and TH within all remaining TH-immunoreactive neurons in the SNc was 67 ± 10.6% for A53T α-synuclein + Nedd4^WT^ and 74.6 ± 6.4% for A53T α-synuclein + Nedd4^C-S^ without a significant difference (p > 0.05, unpaired t-test) between the two animal groups. Stereological analysis of neurons co-labeled with TH and NeuN in the ventral tegmental area (Suppl Fig. 3), which is typically resistant to AAV-mediated α-synuclein-induced neurodegeneration (Chung et al., 2009a, 2009b), did not show a significant difference between cell numbers. These data suggest that in the absence of α-synuclein-induced cell loss, expression of WT or catalytically inactive Nedd4 is not toxic to dopaminergic neurons.

### Nedd4-mediated degradation reduced the accumulation and aggregation of α-synuclein in the SN

To assess whether Nedd4 protects by promoting the clearance of α-synuclein in vivo, we performed serial fractionation and measured the levels of cytosolic and RIPA-insoluble/SDS-soluble α-synuclein in littermate animals. We found that the level of cytosolic α-synuclein was reduced in Nedd4^WT^ compared to Nedd4^CS^ expressing animals, whereas quantification of the RIPA-insoluble/SDS-soluble fraction showed significantly increased gel-excluded and oligomeric forms of α-synuclein (indicative of aggregated protein) in animals expressing Nedd4^CS^ (Fig. 5A). Thus, total α-synuclein levels and preferentially aggregated forms were decreased when wild-type Nedd4 is overexpressed. To determine whether Nedd4 ubiquitinates monomeric as well as aggregated forms of α-synuclein, we reconstituted the ubiquitination of wild-type and A53T mutant α-synuclein in vitro as described previously (Tofaris et al., 2011). We found that the A53T mutant α-synuclein is similarly ubiquitinated to the wild-type protein. Importantly, for the A53T mutant, which is prone to aggregation, we observed that aggregated species present in the reaction before the addition of ubiquitin were also ubiquitinated as evidenced by the shift in their molecular weight following the addition of recombinant ubiquitin (Fig. 5B). Collectively, these data suggest that Nedd4 overexpression can prevent the accumulation of α-synuclein in vivo and may directly promote the clearance of aggregated α-synuclein.

## Discussion

We have provided multiple independent lines of evidence to show that, the ubiquitin ligase Nedd4 protects against α-synuclein-induced toxicity in two animal models of PD. In *Drosophila*, we showed that when Nedd4 activity is enhanced by overexpression, it is sufficient to prevent toxicity from high levels of ectopically expressed wild-type human α-synuclein in the eye or the nervous system whereas endogenous Nedd4 is especially critical for prevention of α-synuclein-induced toxicity in the dopaminergic neurons. In the rat SN, using a complementary approach, we found that expression of wild-type but not inactive Nedd4 decreased degeneration of dopaminergic neurons that is induced by chronic overexpression of A53T mutant human α-synuclein.

A role for Nedd4 in the human condition is supported by the identification of a coding SNP as a risk factor for idiopathic PD (Srinivasan et al., 2009), the finding that Nedd4 mRNA expression is increased in brain regions with Lewy body pathology (Dumitriu et al., 2012) and our neuropathological observations that Nedd4 is up-regulated in a subpopulation of pigmented neurons containing Lewy bodies (Tofaris et al., 2011). Since Nedd4 is a ubiquitin ligase that functions in the endosomal–lysosomal pathway, our data are also consistent with other studies which have implicated this pathway in PD pathogenesis (Dodson et al., 2012) and genetic screens showing that genetic perturbations in endosomal trafficking increase α-synuclein toxicity in relevant cell models (Willingham et al., 2003). A number of observations are consistent with the notion that degradation of α-synuclein by lysosomes is an important pathway in PD pathogenesis: Mutations in glucocerebrosidase (GBA), a lysosomal enzyme linked to Gaucher's disease, are the most common genetic risk factor for sporadic PD (Sidransky et al., 2009). In transgenic models, mutant GBA leads to α-synuclein accumulation in ubiquitinated inclusions whereas virally-mediated overexpression of wild-type GBA is protective (Sardi et al., 2011). Similarly, knockout of the lysosomal protease cathepsin D in mice causes accumulation of α-synuclein in brain whereas cathepsin D overexpression is protective (Cullen et al., 2009). In this context, Nedd4, a ubiquitin ligase that targets proteins to lysosomes, provides an enzymatic link between this striking association of α-synucleinopathy and lysosomal degradation, and given its specificity for only a few protein-substrates, an opportunity for a targeted therapy aimed at preventing the accumulation of α-synuclein in brain. Although Nedd4 has other substrates, especially membrane-bound proteins, activation of this E3 in brain may be well tolerated as evident from our observations on lifespan and those of others that within a certain range of expression in the *Drosophila* nervous system, Nedd4 levels per se do not cause defects in synaptogenesis or axon guidance (Myat et al., 2009; Zhong et al., 2011).

Advancing age is the most important risk factor for developing sporadic PD. In our animal models age-related changes in either phenotype or cell numbers due to α-synuclein expression were modified by ubiquitin-dependent degradation by Nedd4. This finding raises the more general possibility that with age the efficiency of protein ubiquitination rather than degradation may be an important contributor to the development of PD and other neurodegenerative diseases. One possible mechanism is oxidative inactivation of enzymes in the ubiquitin pathway. In line with this suggestion is the finding that parkin, another ubiquitin ligase implicated in autosomal-recessive PD, is oxidatively inactivated in sporadic forms of the disease (Lavoie et al., 2005).

Our data show that the ubiquitin ligase activity of Nedd4 is necessary for its neuroprotective action, which at least in part, is mediated by a direct effect on α-synuclein levels. In *Drosophila* endogenous Nedd4 appears to have a tissue- and cell-specific protective effect: one explanation is that although a 2-fold reduction in endogenous Nedd4 is not sufficient to further increase the levels of ectopically overexpressed α-synuclein as assessed by immunoblotting of whole brain lysates, the rate of α-synuclein clearance is altered in susceptible cell populations in the living brain. The effect of Nedd4 overexpression on α-synuclein levels in the fly and rat brain tissue would be consistent with such explanation. In addition, it is well established that α-synuclein is degraded by multiple pathways (Tofaris et al., 2001, 2011; Webb et al., 2003) which may exhibit variable functional redundancy in neuronal versus non-neuronal tissues. Alternatively, accumulation of other cell-specific substrates could synergistically contribute to α-synuclein-induced dysfunction as Nedd4 RNAi per se is not toxic. It is also noteworthy that Nedd4 is up-regulated in surviving neurons around areas of traumatic brain injury in mice (Sang et al., 2006) and its yeast ortholog can protect against oxidative stress (Hoshikawa et al., 2003) and was previously shown by us to protect against α-synuclein toxicity in the corresponding yeast model (Tofaris et al., 2011). Therefore, an additional, α-synuclein-independent protective effect cannot be excluded. This withstanding, our finding that Nedd4 is protective against human α-synuclein toxicity in two evolutionarily distant model organisms strongly suggests that activation of this conserved ubiquitination pathway should be considered as a target for neuroprotective therapies. In striking confirmation of this prediction, and while our manuscript was under consideration, a genetic drug screen identified a Nedd4 activator as a modifier of α-synuclein toxicity in yeast and iPSc-derived cortical neurons (Tardiff et al., 2013). Previous studies showing that Nedd4 activity is regulated by intra-molecular interactions (Wang et al., 2010) may also be informative in the development of small molecule activators.
