# Supplementary material for: Enhanced ubiquitin-dependent degradation by Nedd4 protects against α-synuclein accumulation and toxicity in animal models of Parkinson's disease
Source: Neurobiol Dis. 2014 Apr;64(100):79–87. doi: 10.1016/j.nbd.2013.12.011 (PMC3988924; doi:10.1016/j.nbd.2013.12.011)
Supplement: Supplementary file 1 — Supplementary figures. [file mmc1.doc]

# Supplementary Figures

**
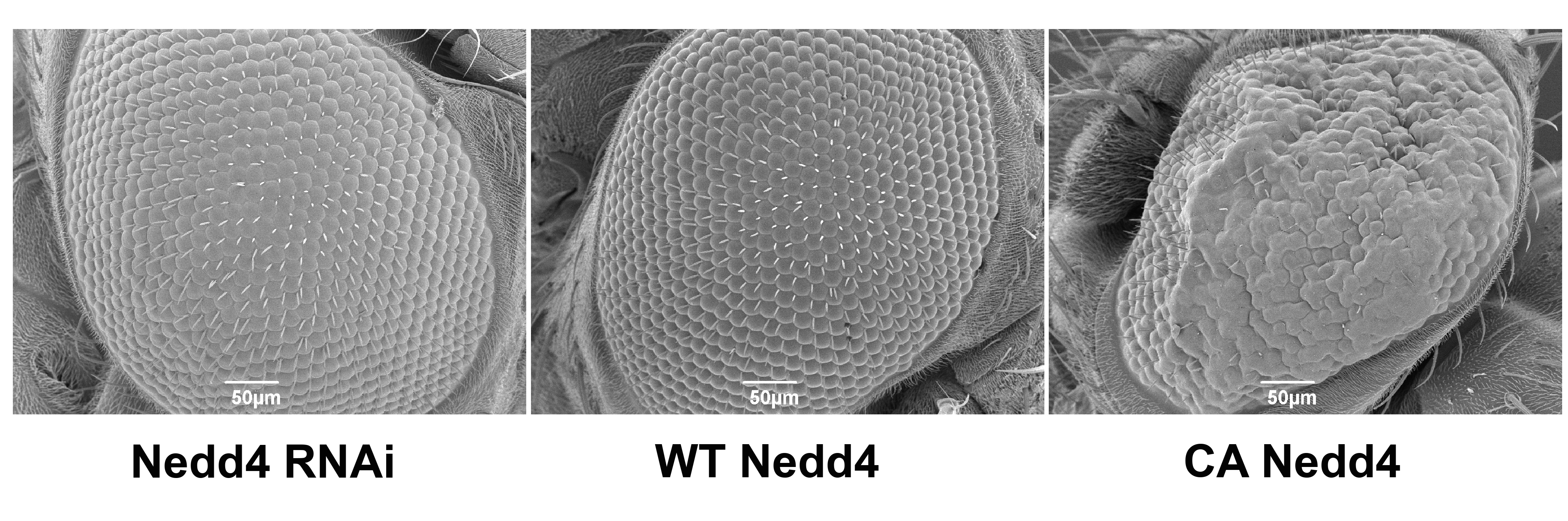
**

**Figure S1: Knockdown or overexpression of Nedd4 in the *Drosophila* eye does not cause a rough eye phenotype whereas expression of C-A inactive Nedd4 is toxic.** Scanning electron microscopy images of fly eyes overexpressing Nedd4 or Nedd4 RNAi did not show a degenerative phenotype at 25 ^o^C or 28 ^o^C. In contrast, expression of catalytically inactive (Cystine to arginine substitution, C-A) Nedd4 caused toxicity, precluding the use of this construct in these toxicity assays. Scale bar, 50μm

**
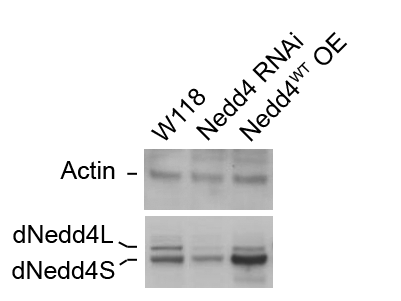
**

**Figure S2: Nedd4 overexpression and knockdown in the nervous system** Immunoblotting of brain homogenates using anti-Nedd4 antibodies recognize two major isoforms in *Drosophila*, a long isoform at 112kDa (dNedd4L) and a short isoform at 92kDa (dNedd4S). When compared to control flies transgenic only for the *elav-GAL4* driver, dNedd4S was successfully overexpressed (lane: Nedd4 OE) and both isoforms were knocked-down (lane: Nedd4 RNAi). After correction for actin loading controls, densitometry showed that RNAi produced a 2-fold reduction in endogenous Nedd4 levels whereas overexpression was of the order of 5-6 fold. It should be pointed out that the effect of RNAi observed by immunoblotting is likely to underestimate the effect *in vivo* as it is diluted by non-neuronal cells, which do not express Nedd4 RNAi.


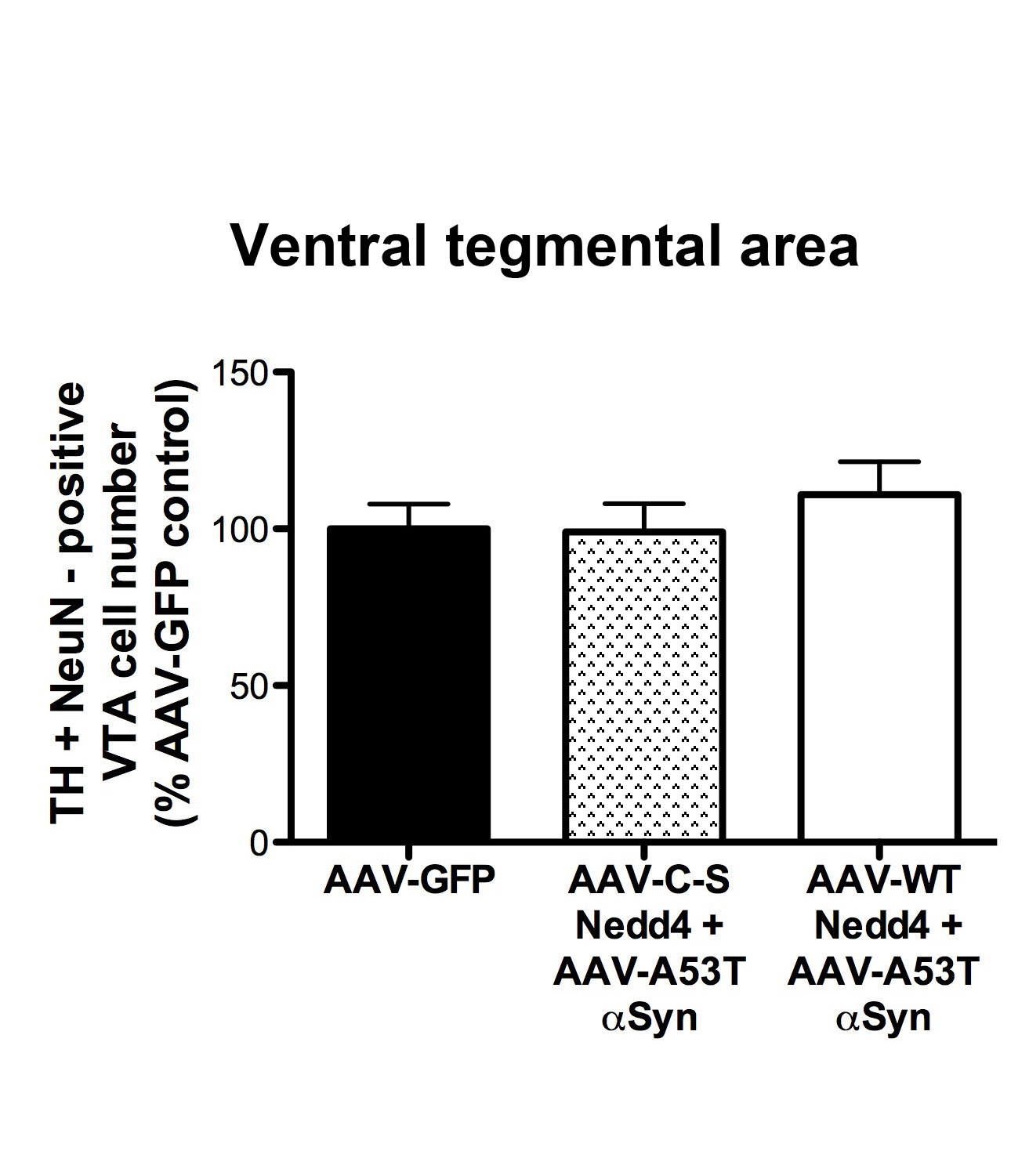


**Figure S3: AAV-mediated overexpression of either WT or CS Nedd4 with A53T α-synuclein** **in the VTA does not cause neurodegeneration.** There was no significant difference in TH neuron counts in the VTA with either WT or C-S Nedd4 overexpression when compared to GFP control.
